# Expression of *Pseudomonas aeruginosa CupD* Fimbrial Genes Is Antagonistically Controlled by RcsB and the EAL-Containing PvrR Response Regulators

**DOI:** 10.1371/journal.pone.0006018

**Published:** 2009-06-23

**Authors:** Helga Mikkelsen, Geneviève Ball, Caroline Giraud, Alain Filloux

**Affiliations:** 1 Imperial College London, Division of Cell and Molecular Biology, Centre for Molecular Microbiology and Infection, South Kensington Campus, London, United Kingdom; 2 Laboratoire d'Ingénierie des Systèmes Macromoléculaires, UPR9027, CNRS-IBSM, Marseille, France; Baylor College of Medicine, United States of America

## Abstract

*Pseudomonas aeruginosa* is a gram-negative pathogenic bacterium with a high adaptive potential that allows proliferation in a broad range of hosts or niches. It is also the causative agent of both acute and chronic biofilm-related infections in humans. Three *cup* gene clusters (*cupA-C*), involved in the assembly of cell surface fimbriae, have been shown to be involved in biofilm formation by the *P. aeruginosa* strains PAO1 or PAK. In PA14 isolates, a fourth cluster, named *cupD*, was identified within a pathogenicity island, PAPI-I, and may contribute to the higher virulence of this strain. Expression of the *cupA* genes is controlled by the HNS-like protein MvaT, whereas the *cupB* and *cupC* genes are under the control of the RocS1A1R two-component system. In this study, we show that *cupD* gene expression is positively controlled by the response regulator RcsB. As a consequence, CupD fimbriae are assembled on the cell surface, which results in a number of phenotypes such as a small colony morphotype, increased biofilm formation and decreased motility. These behaviors are compatible with the sessile bacterial lifestyle. The balance between planktonic and sessile lifestyles is known to be linked to the intracellular levels of c-di-GMP with high levels favoring biofilm formation. We showed that the EAL domain-containing PvrR response regulator counteracts the activity of RcsB on *cupD* gene expression. The action of PvrR is likely to involve c-di-GMP degradation through phosphodiesterase activity, confirming the key role of this second messenger in the balance between bacterial lifestyles. The regulatory network between RcsB and PvrR remains to be elucidated, but it stands as a potential model system to study how the equilibrium between the two lifestyles could be influenced by therapeutic agents that favor the planktonic lifestyle. This would render the pathogen accessible for the immune system or conventional antibiotic treatment.

## Introduction

Most microorganisms preferentially live in their environment as multicellular communities in association with inert or biological surfaces [1]. These populations, called biofilms, are encased in a matrix of exopolysaccharides, which contributes to the mechanical and biological properties of the community. The attachment to a surface can prevent cells from being swept away by liquid flows, and thus provides an efficient strategy for persistence in a chosen and favorable environment. Moreover, bacteria in biofilms have evolved new biological capabilities that make them resistant to toxic agents and antibiotics, as well as to the immune system. The development of such resistance is of particular importance in the context of bacterial pathogens establishing a biofilm in the host during infection [2]. The formation of the biofilm results in persistence and chronic infections, since they are rarely eradicated by conventional antimicrobial treatments.

*Pseudomonas aeruginosa* is a potent opportunistic pathogen, which proliferates within a wide variety of hosts from amoeba to mammals, including nematodes, insects and plants [3]–[5]. *P. aeruginosa* persistent infections can be the result of biofilm formation on medical devices, such as catheters or prostheses in the case of hospitalized patients, or within the lungs and respiratory tract of cystic fibrosis patients [2], [6]. *In vitro* studies have shown that the biofilm developmental process follows a number of steps, from the initial attachment to a surface to the elaboration of the mature and resistant biofilm structure [7]. At each step, dedicated molecular determinants are involved. Several of these determinants are cell surface appendages that promote mobility towards the surface, initial attachment to or motility along the surface, and the formation of microcolonies .

The *P. aeruginosa* Cup fimbriae represent one class of these appendages, and they are assembled at the bacterial cell surface through the “chaperone-usher pathway” after which they were named [8]–[10]. The fimbrial structure results from the multimerisation of the major fimbrial subunit, which forms the rod. In some cases, a tip structure is connected to the rod, and this allows presentation of an adhesin at the far end of the fiber, which mediates specific binding [11]. Analysis of the PAO1 genome [12] revealed that multiple copies of *cup* gene clusters, *cupA*, *cupB* and *cupC*, could be identified [8]. The organization of these *cup* clusters varies, and it is not yet clear what their respective function is in bacterial attachment and/or biofilm formation. They might play a role at different stages in the process, or they could be specialized to different hosts or environmental niches. This is supported by the observation that their expression is tightly and differentially controlled. The *cupA* gene expression is rather complex, involving the H-NS-like MvaT transcriptional regulator that represses phase variable expression of these genes [13]–[14]. In addition, it was shown that several regulators positively control *cupA* gene expression under anaerobic conditions [15]. The *cupB* and *cupC* genes are controlled by a two-component regulatory system (RocA1-RocS1-RocR), which involves two response regulators RocA1 and RocR, and an unorthodox sensor RocS1 [16]. Whereas RocA1 positively controls *cupB* and *cupC* gene expression, RocR down-regulates their transcription. Importantly, RocR does not bind DNA but displays a phosphodiesterase activity linked to an EAL domain [16]–[17]. Diguanylate cyclases (GGDEF domain-containing proteins) and EAL-containing proteins are enzymes involved in the maintenance of cellular concentration of the second messenger cyclic-di-GMP (c-di-GMP) [18]. GGDEF domains catalyze the formation of the c-di-GMP, while EAL domains are responsible for its hydrolysis into 5′pGpG. c-di-GMP appears as a central signaling molecule in the process of biofilm formation, with high intracellular levels directing bacteria towards a sessile lifestyle [19]–[20]. The phosphodiesterase activity of RocR, combined with the down regulation of *cupB* and *cupC* genes and the decreased in biofilm level, is in agreement with this model.

**Figure 1:**
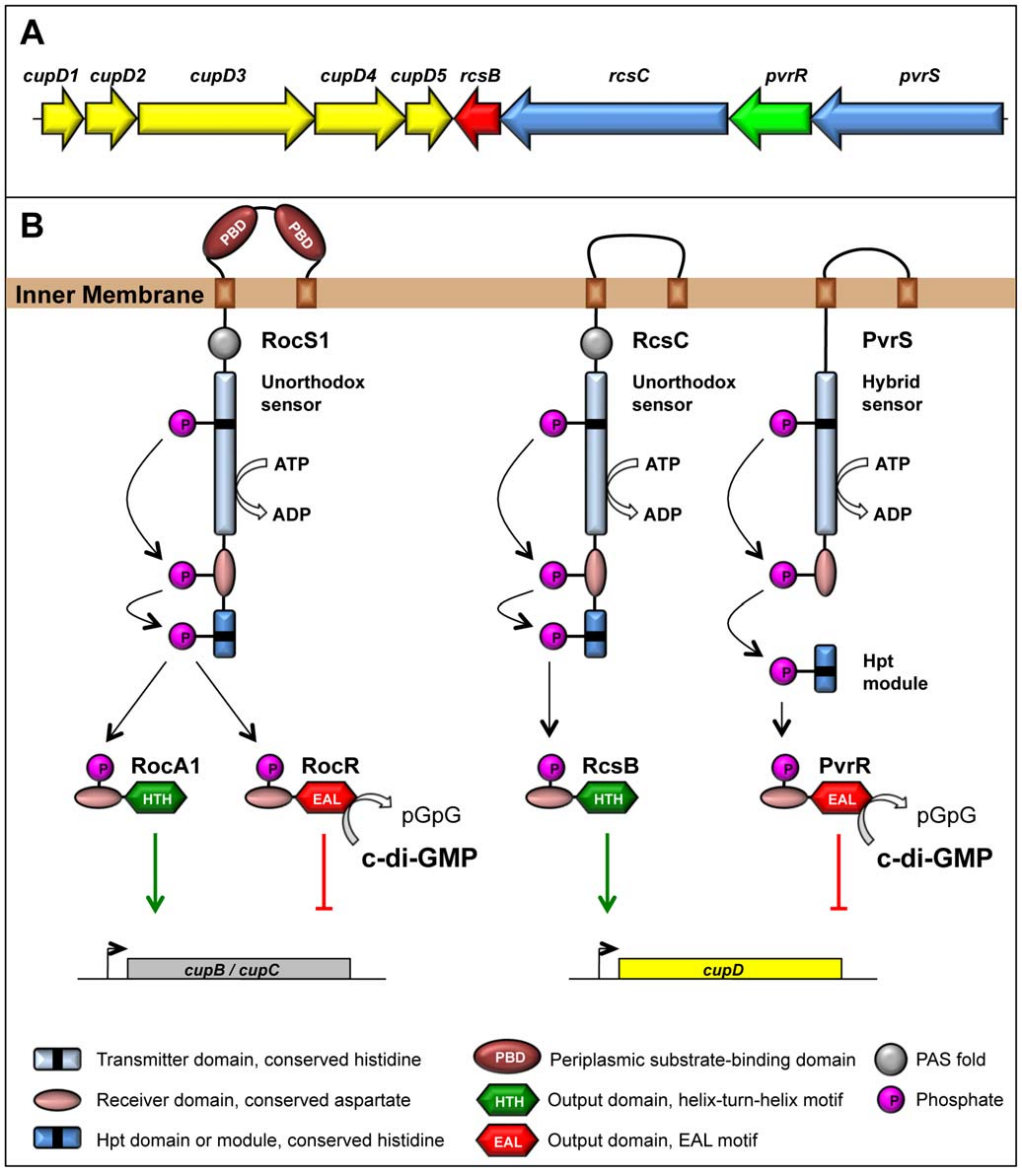
Genetic organization and proposed signaling mechanism. A) Genetic organization of the *cupD/rcs/pvr* locus. *cupD1*, *cupD2* and *cupD3* encode the major fimbrial subunit, a chaperone and an usher respectively, whereas *cupD4* and *cupD5* encode a putative adhesin and a second chaperone. *rcsC* and *pvrS* encode putative two-component sensors, and *rcsB* and *pvrR* are predicted to encode their cognate response regulators. B) Left: Domain organization and signaling mechanism of the RocS1/A1/R two-component system that controls expression of the *cupB* and *cupC* gene clusters. Right: Domain organization and proposed signaling mechanism of the Rcs and Pvr two component systems that control expression of the *cupD* gene cluster. RocS1 and RcsC are unorthodox sensors with a transmitter domain, a receiver domain and an Hpt phosphorelay. PvrS is a hybrid sensor that requires an external Hpt module to relay the signal to the response regulator. Like RocA1, RcsB is likely to activate transcription directly *via* its helix-turn-helix motif that allows DNA binding. Conversely, RocR and PvrR are likely to repress transcription indirectly *via* the EAL domain, which degrades the second messenger cyclic-di-GMP.

Whereas *P. aeruginosa* is the ultimate opportunistic pathogen, the level of virulence can vary greatly from one isolate to another. Some isolates carry additional genomic blocks inserted into the core genome, also known as pathogenicity islands that are likely acquired by lateral gene transfer. Usually these genetic islands contribute to virulence by providing additional pathogenic functions. The *P. aeruginosa* PA14 strain is a clinical isolate and a much more virulent strain than PAO1 [21], despite having the same host range. Two pathogenicity islands, PAPI-1 and PAPI-2, have been identified in PA14 [22]. In particular, PAPI-1 is 108 kb-long and is entirely absent from the PAO1 genome. It carries many genes of unknown function. However, a *cup* gene cluster, annotated *cupD*, and a set of genes encoding proteins of two-component regulatory systems (Figure 1A), were found clustered together and flanked by two direct repeats, which suggests that they have been acquired simultaneously. Among the genes encoding the two-component systems, *pvrR* has previously been shown to encode a “phenotype variant regulator” involved in regulating the frequency of antibiotic resistant variants [23]. In addition, two other genes encoding a response regulator and a histidine kinase sensor were annotated *rcsB* and *rcsC*, respectively (http://www.pseudomonas.com). They were named after the sequence homology they display with other two-component regulatory systems from *Salmonella enterica* [24] or *Escherichia coli* [25]. In these species, the Rcs system is a “regulator of capsule synthesis”, which is the Vi polysaccharide for *S. enterica* and colanic acid for *E. coli*. To our current knowledge, the *P. aeruginosa* Rcs system has not been shown to be involved in the control of expolysaccharide production.

In the present study we aimed to understand whether the two-component regulatory systems Pvr and Rcs are involved in controlling *cupD* gene expression, and whether they act in a synergistic or antagonist manner. Furthermore, we investigated whether *cupD* gene expression resulted in biofilm-associated phenotypes. Interestingly, we found that CupD fimbrial assembly provides the bacterium with a number of biological properties, which are not strictly similar to those conferred by the other characterized Cup systems.

## Results

### Characterization of the *cupD-rcsBC-pvrRS* gene cluster

The five *cupD*-genes, *cupD1-5*, are organized in a similar manner as the previously described *cupA* system from *P. aeruginosa* PAO1 (Figure 1A) [8] with *cupD1, cupD2* and *cupD3* encoding the major fimbrial subunit, a chaperone, and the outer membrane usher, respectively. *cupD4* and *cupD5* are predicted to encode an adhesin and a second chaperone (Figure 1A and Table 1). Located adjacent to the *cupD* genes, and organized in the opposite orientation, are four genes encoding two-component systems: two sensors and two response regulators (Figure 1 and Table 1). *rcsB* encodes a response regulator with an output domain containing a helix-turn-helix (HTH) motif, suggesting that it binds DNA and functions as a transcriptional regulator [26]. *pvrR* encodes a second response regulator, which contains an EAL-domain that is likely to be involved in degradation of the second messenger c-di-GMP [19], [23], [27]. The putative cognate sensor for *rcsB* is the unorthodox sensor *rcsC*, whereas the putative cognate sensor for *pvrR* is a hybrid sensor. All these genes are clearly annotated on the PA14 genome sequence (http://www.pseudomonas.com), apart from the hybrid sensor, which we propose to name *pvrS*.

**Table 1:** Characterization of the *cupD/rcs/pvr* gene cluster.

**PA14-annotation number** *	59710	59720	59735	59750	59760	59770	59780	59790	59800
**Gene name**	*cupD1* *	*cupD2* *	*cupD3* *	*cupD4* *	*cupD5* *	*rcsB* *	*rcsC* *	*pvrR* *	*pvrS* **
**Size ORF (bp)**	549	747	2614	1347	717	696	3255	1200	2796
**No amino acids**	182	248	870	448	238	231	1084	399	931
**Protein characteristics**	Fimbrial domain	Pili assembly	Usher	Fimbrial domain	Chaperone	Receiver domain HTH-domain	Histidine kinase H-ATPase Receiver domain Htp domain	Receiver domain EAL-domain	Histidine kinase H-ATPase Receiver domain
**Predicted function**	Major fimbrial subunit	Chaperone	Usher	Adhesin	Chaperone	Two-component response regulator	Two-component sensor kinase	Two-component response regulator	Two-component sensor kinase

* Annotation according to http://www.pseudomonas.com.

** Proposed gene name for PA14_59800.

The organization of the Rcs/Pvr system is highly reminiscent of the Roc system that controls *cupB* and *cupC* expression in *P. aeruginosa* PAO1 (Figure 1B) [16]. Transcription of *cupB* and *cupC* genes is activated by the HTH-containing response regulator RocA1 and repressed by the RocR response regulator, which contains an EAL domain [17].

Cup fimbriae have previously been shown to be important for biofilm formation in *P. aeruginosa* [8]–[10], [16], [28]. In order to assess the influence of *cupD* and the two-component systems on biofilm formation, a series of mutants were constructed by deletion of *cupD1-5*, *rcsB, rcsC, pvrR* or *pvrS*, and biofilm formation of wild type and mutant strains was assessed in 24-well plates using crystal violet (CV) staining as described in Materials and Methods. We showed that none of these mutations had any effect on biofilm formation (Figure S1). One reason for this could be that the *cupD* genes are not expressed in standard laboratory conditions, as shown for other *cup* genes [8]–[9], [16], and that Cup fimbrial assembly requires activation of a cognate two-component system [9], [16]. Based on this observation, we hypothesized that *cupD* gene expression in *P. aeruginosa* PA14 requires expression of the response regulator RcsB.

### RcsB positively controls *cupD* gene expression

**Figure 2:**
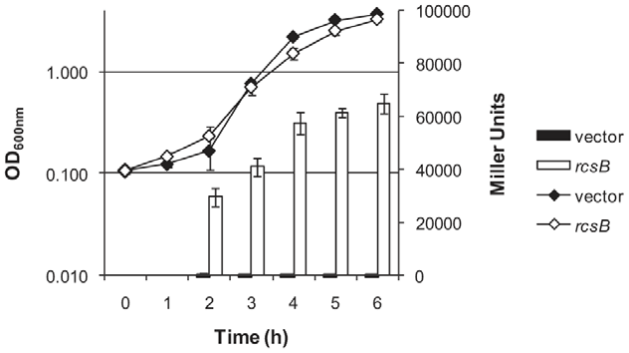
Overexpression of *rcsB* induces *cupD* gene expression. Growth and β-galactosidase activity of PA14::*cupD1-lacZ*/pBBR1MCS-5 (black diamonds and bars, respectively) and PA14::*cupD1-lacZ*/pBBR1MCS-5-*rcsB* (white diamonds and bars, respectively).

A DNA fragment that contained 530 bp upstream from the *cupD1* gene and included the putative promoter region of the *cupD* gene cluster, was PCR-amplified and cloned into miniCTX-lacZ, a vector for engineering *lacZ* reporter gene fusions that can be integrated into the *P. aeruginosa* genome [29]. The *cupD1-lacZ* promoter fusion was thus integrated on the chromosome of the PA14 strain, yielding PA14::*cupD1-lacZ*, as described in Materials and Methods (Table 2). Furthermore, the *rcsB* gene was cloned under the control of the *lac* promoter into the broad host range vector pBBR1MCS-5 and introduced into PA14::*cupD1-lacZ*. The transconjugants appeared blue on LB-agar plates supplemented with X-gal (5-bromo-4-chloro-3-indolyl-beta-D-galactopyranoside), whereas the PA14::*cupD1-lacZ* strain carrying the empty vector remained white, indicating that expression of the *cupD1-lacZ* fusion only occurred upon RcsB overproduction. The activation of the *cupD1-lacZ* fusion in the *rcsB-*overexpressing strain was quantified by performing a β-galactosidase liquid assay, as described in Materials and Methods (Figure 2). RcsB-dependent induction of the *cupD1-lacZ* fusion resulted in a signal approaching 70,000 Miller Units, compared to around 100 Miller Units for the vector control. This result was a strong indication that the response regulator RcsB positively controls the expression of the *cupD* gene cluster.

**Table 2:** Bacterial strains and plasmids.

Strain/Plasmid	Genotype/Description	Source
***Escherichia coli***		
TG1	*supE* Δ(*lac-proAB*) *thi hsdR*Δ*5* (F' *traD36 rpoA^+^B^+^ lacI* ^q^ *Z*ΔM15)	Lab collection
OmniMAX	F′ {*proAB* ^+^ *lacI* ^q^ *lacZ*ΔM15 Tn10(*Tet* ^r^) Δ (*ccdAB*)} *mcrA* Δ (*mrr-hsd*RMS-*mcr*BC) ϕ80(*lacZ*) ΔM15 Δ(*lacZ*YA-*arg*F) U169 *end*A1 *rec*A1 *sup*E44 *thi*-1 *gyr*A96 *rel*A1 *ton*A *pan*D	Invitrogen
SM10 (λpir)	*leu*, *tonA*, *lacY*, *supE*, recA::RP4-2-Tc::Mu, Km^r^, λpir	Lab collection
1048	pRK2013, ColE1 ori tra^+^ mob^+^ Km^r^. Helper strain for conjugation of plasmids	Lab collection
TOP10F'	F' {*lacI* ^q^ Tn10 (*Tet* ^r^)} *mcrA* Δ(*mrr*-*hsd*RMS-*mcr*BC) Φ80*lacZ*ΔM15 Δ*lac*χ74 *recA*1 *araD*139 Δ(*ara-leu*)7697 *galU galK rpsL* (Str^r^) *endA*1 *nupG*	Invitrogen
CC118 (λpir)	Host strain for pKNG101 replication	Lab collection
BL21(DE3)	F̅*ompTgal* [*dcm*] [*lon*] hsdSB (r̅k m B̅) B strain with the DE3 propage carrying the T7 RNA polymerase gene	Lab collection
***Pseudomonas aeruginosa***		
PA14	Wild type	[34]
PAO1	Wild type	Lab collection
PA14::*cupD1-lacZ*	Wild type with a *cupD1-lacZ* fusion integrated on the chromosome	This study
PA14Δ*cupD*	PA14 with the entire *cupD* gene cluster deleted (*cupD1-5*)	This study
PA14Δ*rcsB*	PA14 with a deletion in *rcsB*	This study
PA14Δ*rcsC*	PA14 with a deletion in *rcsC*	This study
PA14Δ*pvrR*	PA14 with a deletion in *pvrR*	This study
PA14Δ*pvrS*	PA14 with a deletion in *pvrS*	This study
PA14::*rcsC*	PA14 mutant (PA Mr-nr-mas-05-3) with a transposon insertion in *rcsC*	[34]
PA14::*rcsB*::*cupD1-lacZ*	PA14 mutant (PA Mr-nr-mas-10-2) with a transposon insertion in *rcsB* and a *cupD1-lacZ* fusion integrated on the chromosome	This study
PA14::*rcsC*::*cupD1-lacZ*	PA14 mutant (PA Mr-nr-mas-05-3) with a transposon insertion in *rcsC* and a *cupD1-lacZ* fusion integrated on the chromosome	This study
PA14::*pvrR*::*cupD1-lacZ*	PA14 mutant (PA Mr-nr-mas-08-1) with a transposon insertion in *pvrR* and a *cupD1-lacZ* fusion integrated on the chromosome	This study
PA14::*pvrS*::*cupD1-lacZ*	PA14 mutant (PA Mr-nr-mas-05-01) with a transposon insertion in *pvrS* and a *cupD1-lacZ* fusion integrated on the chromosome	This study
**Plasmids**		
pKNG101	Suicide vector for gene replacement in *P. aeruginosa*. *SacB*, Sm^r^	[48]
pFLP2	Source of Flp recombinase, Ap^r^. For unmarked integration into the *P. aeruginosa* chromosome.	[49]
pBBR-MCS-4	Broad host range vector, Ap^r^	[50]
pBBR-MCS-4-*pvrR*	*pvrR* cloned into pBBR-MCS-4 (SmaI)	This study
pBBR-MCS-5	Broad host range vector, Gm^r^	[50]
pBBR-MCS-5-*rcsB*	*rcsB* cloned into pBBR-MCS-5 (HindIII/XbaI)	This study
pBBR-MCS-5-*pvrR*	*pvrR* cloned into pBBR-MCS-5 (KpnI/XbaI)	This study
miniCTX-*lacZ*	Vector for unmarked integration into *P. aeruginosa att* site. *oriT*, FRT, *int*, Tc^r^	[29]
miniCTX-*cupD1-lacZ*	*cupD1* promoter cloned into miniCTX-lacZ (SmaI)	This study
pCR2.1-TA	TA cloning vector for PCR products. *lacZ*, ColE1 f1 ori Ap^r^ Km^r^	Invitrogen
pCR2.1-*cupD1*-6H	pCR2.1 carrying the *cupD1* gene with an C-terminal hexahistidine tag (6H)	This study

### *rcsB* expression results in assembly of CupD fimbriae

**Figure 3:**
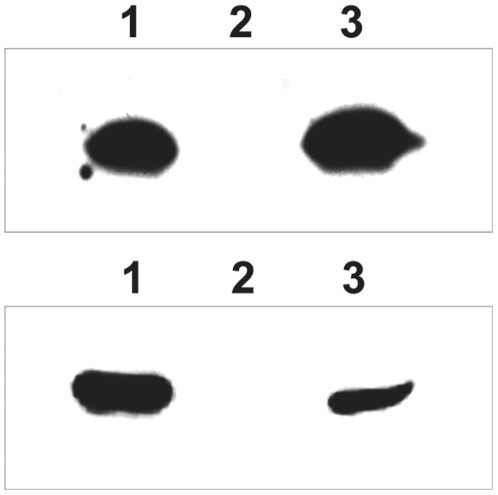
Overexpression of *rcsB* leads to the production of CupD1 and assembly of CupD fimbriae. Western blot of fimbrial protein using a CupD1 antibody. 1) PA14/pBBR1MCS-5-*rcsB*, 2) PA14/pBBR1MCS-5 and 3) PA14::*rcsC*. Upper panel: Whole cell extract. Lower panel: Sheared extracellular fimbriae as described in Materials and Methods.

We next wanted to investigate if RcsB-dependent induction of the *cupD* genes would lead to the production and assembly of CupD fimbriae. We have previously shown that Cup fimbrial assembly at the surface can be monitored by shearing of cell surface appendages upon gentle agitation [9]–[10]. Proteins from whole cell extracts, as well as from sheared extracellular fimbriae, were therefore prepared as described in Materials and Methods. Proteins were separated on SDS-PAGE gels and visualized by Western immunoblotting using an antibody directed against CupD1, the major subunit of the CupD fimbriae. In the PA14 strain overexpressing *rcsB*, clear bands could be detected at the predicted size for CupD1 (18 kDa) in both the whole cell and the sheared fractions (Figure 3). Conversely, no CupD1 was detected in PA14 carrying the empty cloning vector. Taken together, this shows that *cupD* genes are not expressed under normal laboratory conditions, but that their transcription is activated upon production of the RcsB response regulator, and this leads to the production of CupD fimbriae, which are assembled on the cell surface.

### *rcsB* overexpression results in multiple phenotypes

**Figure 4:**
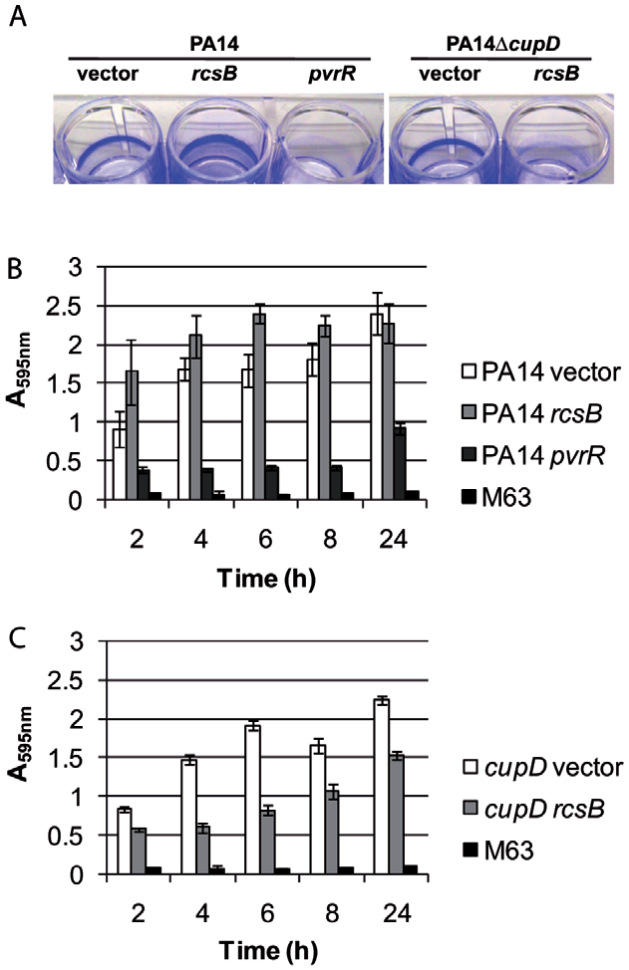
Biofilm formation phenotypes in PA14 and derivative strains. Biofilms were grown in 24 well plates and stained with crystal violet (CV). A) CV stained biofilms after 6 h incubation. PA14 or PA14Δ*cupD* carrying pBBR1MCS-5 (vector), pBBR1MCS-5-*rcsB* (*rcsB*) or pBBR1MCS-4-*pvrR* (*pvrR*) (only for PA14) are shown. B) and C) Quantification of biofilm formation at different times (2 to 24 hours) using crystal violet staining. PA14 wild type (B) or PA14Δ*cupD* (C) carrying pBBR1MCS-5 (white bars), pBBR1MCS-5-*rcsB* (light grey bars) and pBBR1MCS-5-*pvrR* (dark grey bars, only for PA14). M63 is a negative control containing cell free M63 medium (black bars).

Since we showed that *rcsB* overexpression resulted in CupD fimbrial assembly, we further tested phenotypes that are known to be associated with the expression of Cup fimbriae. Firstly, we tested the biofilm phenotype by investigating the attachment of PA14 and derivative strains to inert surfaces using 24-well polystyrene plates and CV staining. We observed that *rcsB-*overexpression in PA14 leads to increased staining compared with the strain carrying the empty vector control (Figure 4A and 4B). Interestingly, the staining pattern was different for the PA14 *rcsB-*overexpressing strain in that it formed a broader and often less distinct ring around the well than the corresponding vector control (Figure 4A). Instead, there was an increased staining of the sides and bottom of the well, suggesting that these cells attached to the whole surface they were in contact with and not only at the air-liquid interface of the culture as usually seen with *P. aeruginosa*.

**Figure 5:**
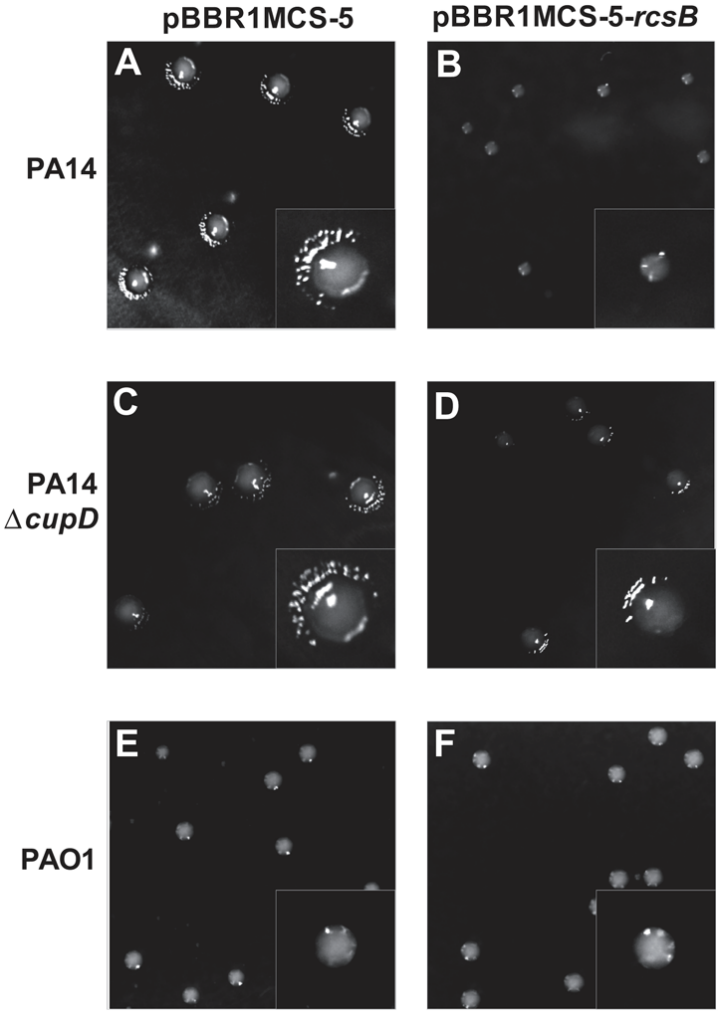
*rcsB* overexpression leads to a small colony morphotype in PA14. PA14 (A and B), PA14Δ*cupD* (C and D) or PAO1 (E and F) strains freshly conjugated with pBBR1MCS-5 (A, C and E) or pBBR1MCS-5-*rcsB* (B, D and F) and plated on *Pseudomonas* isolation agar supplemented with appropriate antibiotics. Images are presented at the same scale. Insets: Enlarged image of a typical colony from each strain.

We also noted that overproduction of RcsB led to a small colony phenotype. Indeed, whereas colonies of PA14 carrying the pBBR1MCS-5 vector had a normal size with wrinkly spreading edges (Figure 5A), the same strain carrying pBBR1MCS-5-*rcsB* gave rise to much smaller colonies with smooth edges (Figure 5B). The colony appearance was not due to any substantial growth defect as determined in M63 liquid culture (Figure 2). This is also in agreement with previous reports on the occurrence of *P. aeruginosa* small colony variants (SCV) upon expression of the *cupA* gene cluster and assembly of CupA fimbriae [30].

**Figure 6:**
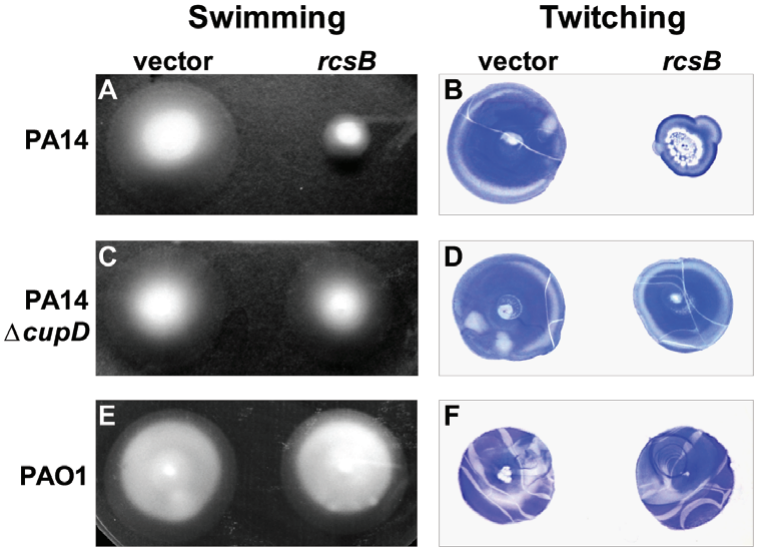
*rcsB* overexpression reduces motility in PA14. Swimming (A, C and E) and twitching (B, D and F) motility of *P. aeruginosa* PA14 (A and B), PA14Δ*cupD* (C and D) or PAO1 (E and F) strains carrying either pBBR1MCS-5 (vector) or pBBR1MCS-5-*rcsB* (*rcsB*). Twitching zones have been visualized using crystal violet staining.

We also investigated the motility phenotypes, since increased biofilm development is likely to be concomitant with a decrease in motility [31]. In agreement with this, we observed that overexpression of *rcsB* in PA14 led to a decrease in flagellar-dependent swimming motility and in type IV pilus-dependent twitching motility (Figure 6A and B).

**Figure 7:**
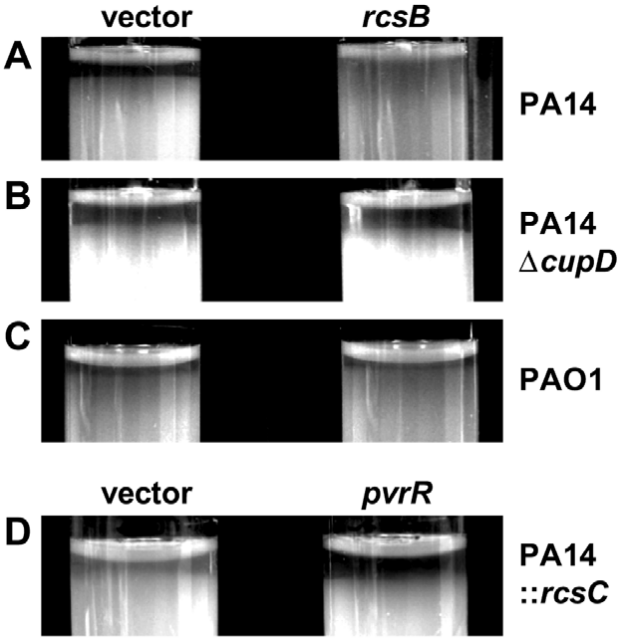
Sedimentation of bacterial cells. *P. aeruginosa* cultures, normalized for OD_600 nm_, were incubated at room temperature without shaking for 24 hours. PA14 (A), PA14Δ*cupD* (B) and PAO1 (C) carry pBBR1MCS-5 (vector) or pBBR1MCS-5-*rcsB* (*rcsB*), while PA14::*rcsC* (D) carries pBBR1MCS-4 (vector) or pBBR1MCS-4-*pvrR* (*pvrR*).

Previous studies have shown that small rough colonies of *P. aeruginosa* 57RP, which are hyper-biofilm formers and affected in all type of motilities, also displayed an autoaggregative phenotype in liquid culture [31]. In order to investigate whether *rcsB* overexpression also resulted in an auto-aggregation phenotype, a sedimentation assay was carried out as previously described [32]–[33]. Briefly, strains were grown in liquid culture supplemented with antibiotics, OD_600 nm_ was adjusted to 2.5 and cells were left to sediment at room temperature for 24 hours. Sedimentation was assessed by the occurrence of a clearing zone at the top of the culture. Surprisingly, the clearing zone was reduced in the *rcsB-*overexpressing strain compared with the vector control, indicating that these cells were impaired in sedimentation (Figure 7A). Impairment in sedimentation could be linked with weakened cell-cell interactions, which could either suggest that the assembly of CupD fimbriae masks components involved in this mechanism, or that CupD fimbrial assembly modifies the physiochemical properties of the cell surface.

### The RcsB-dependent phenotypes are linked to CupD fimbrial assembly

RcsB overproduction results in CupD fimbrial assembly and subsequently in a number of associated phenotypes, as described in the previous paragraph. We investigated whether each of the phenotypes observed was directly linked to the presence of CupD fimbriae. The *rcsB* overexpressing plasmid (pBBR1MCS-5-*rcsB*) was thus introduced into the *cupD* mutant, PA14Δ*cupD*, and all phenotypes were assessed and compared to the same strain carrying the empty vector. In the *cupD* genetic background, most of the RcsB-induced phenotypes were either abolished or greatly reduced, including sedimentation (Figure 7B), motility (Figure 6C and D) and colony morphology (Figure 5C and D). Indeed, the colonies of the mutant overexpressing *rcsB* were slightly smaller than those of PA14 carrying the empty vector, but they were not as small as PA14 overexpressing *rcsB*, and a rough edge was still visible. Surprisingly, when looking at biofilm formation, the increased biofilm expression linked to *rcsB* overexpression was not only lost in the PA14Δ*cupD1* mutant, but the attachment to the inert surfaces of the polystyrene plates was drastically reduced when compared to PA14Δ*cupD1* carrying the empty vector (Figures 4A and 4C). This could either suggest that *rcsB* overexpression prevents PA14 biofilm formation, or that it results in biofilm detachment when CupD fimbriae are lacking. In the presence of CupD fimbriae, the unknown component or process that interferes with PA14 biofilm formation is masked or inactive, and CupD fimbriae promote biofilm formation.

In order to assess whether the RcsB-dependent phenotypes were strictly dependent on genes carried on the PA14 pathogenicity islands, we overexpressed *rcsB* in the *P. aeruginosa* PAO1 strain, in which PAPI-1 and PAPI-2 are absent [22]. The PAO1 colony morphology (Figure 5E and F), motility (Figure 6E and F) and sedimentation (Figure 7C) properties were not affected by *rcsB* overexpression. Moreover, overexpression of *rcsB* in PAO1 did not result in decreased biofilm formation, as seen in the *cupD* mutant (data not shown), suggesting that the underlying causes of the observed phenotypes are specific to PA14.

### PvrR acts antagonistically to RcsB

As seen in Figure 1A, the gene encoding the RcsB response regulator is linked with other genes encoding proteins of two-component regulatory systems. The gene encoding the EAL-domain containing response regulator PvrR is of particular interest, since it is very similar to the RocR response regulator, which down-regulates *cupB* and *cupC* gene expression [16], [23]. We wished to test whether PvrR could down-regulate *cupD* gene expression, and thus counteract the positive control exerted by RcsB. Since the level of *cupD* gene expression is barely detectable in standard laboratory growth conditions (Figure 2), a strain had to be engineered that overexpressed the *cupD* genes to assess a possible repression by PvrR. As shown above, introducing pBBR1MCS-5-*rcsB* is sufficient to obtain *cupD* gene expression (Figure 2), but to avoid constructing strains carrying multiple plasmids we made use of a fortuitous observation. We integrated the *cupD1-lacZ* reporter fusion into the chromosome of various mutants of the available PA14 transposon mutant library [34] (Table 2) and observed that *cupD1* gene expression was induced in the PA14::*rcsC*::*cupD1-lacZ* strain, although to a lesser extent than in PA14::*cupD1-lacZ* carrying pBBR1MCS-5-*rcsB*. Moreover, we also observed that *cupD* gene expression in PA14::*rcsC*::*cupD1-lacZ* resulted in CupD fimbriae assembly as shown by immunoblot analysis of sheared cell fraction (Figure 3). In the PA14::*rcsC*::*cupD1-lacZ* strain, the MAR2xT7 transposon is inserted in *rcsC*, which encodes the putative cognate sensor for the RcsB response regulator. Although the observation was unexpected, we reasoned that *cupD* gene expression in this mutant probably resulted from *rcsB* expression driven from a promoter located within the transposon. This interpretation is likely since we have shown in this study that *rcsB* overexpression is sufficient to drive *cupD* gene expression.

**Figure 8:**
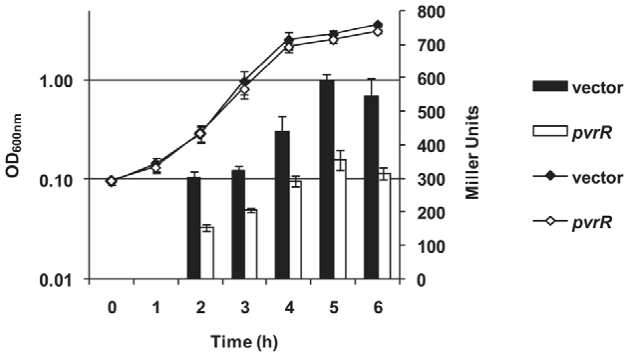
Overexpression of *pvrR* reduces *cupD* gene expression. Growth and β-galactosidase activity of PA14::*rcsC*::*cupD1-lacZ*/pBBR1MCS-4 (black diamonds and bars, respectively) and PA14::*rcsC*::*cupD1-lacZ*/pBBR1MCS-4-*pvrR* (white diamonds and bars, respectively).

We then cloned the *pvrR* gene into the broad host range vector pBBR1MCS-4, yielding pBBR1MCS-4-*pvrR* (Table 2), and introduced it into PA14::*rcsC*::*cupD1-lacZ*. The PA14::*rcsC*::*cupD1-lacZ* strain overexpressing *pvrR* was less blue on LB-agar plates containing X-gal, as compared with the same strain carrying the vector control. The activity of the *cupD-lacZ* fusion was analyzed by measuring β-galactosidase activity in liquid assay, which confirmed an approximately 2-fold decrease in Miller units when *pvrR* was expressed (Figure 8).

**Figure 9:**
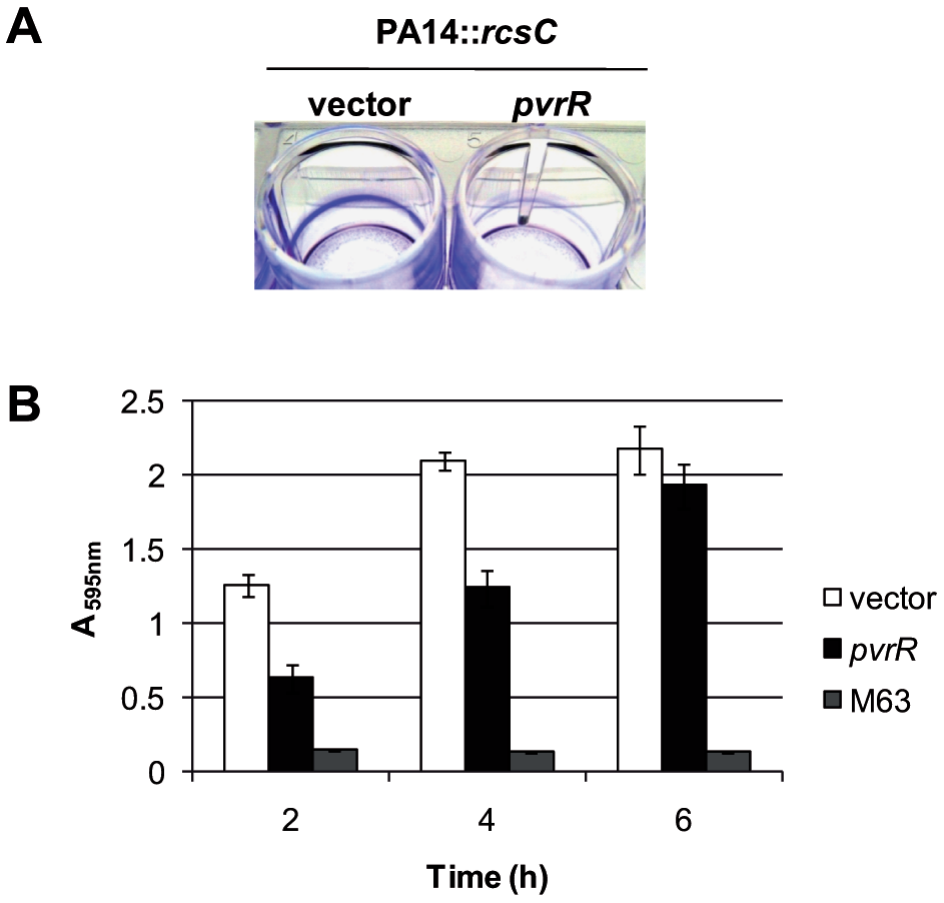
*pvrR* overexpression reduces biofilm formation. Biofilms of PA14::*rcsC* carrying either the empty vector pBBR1MCS-4 (vector) or the *pvrR*-overexpressing plasmid pBBR1MCS-4-*pvrR* (*pvrR*) were analyzed. A) Image of crystal violet stained biofilms after 4 hours. B) Quantification of crystal violet stained biofilms at different times of growth (2–6 hours). PA14::*rcsC* carrying pBBR1MCS-4 (white bars) and pBBR1MCS-4-*pvrR* (black bars) are shown. M63 is a negative control containing cell free M63 medium (dark grey bars).

We also analyzed whether *pvrR* expression could revert the RcsB-dependent phenotypes and observed that introduction of pBBR1MCS-4-*pvrR* into PA14 (Figure 4A and 4B) or PA14::*rcsC* (Figure 9) decreased biofilm formation. We also observed that the sedimentation phenotype of the PA14::*rcsC* strain (Figure 7D) was similar to the one observed with PA14 carrying pBBR1MCS-5-*rcsB* (Figure 7A). However, introduction of pBBR1MCS-4-*pvrR* in PA14::*rcsC* reverted the RcsB-dependent sedimentation phenotype (Figure 7D) to the phenotype displayed by PA14 carrying the empty vector (Figure 7A).

Overall, our data show that PvrR acts antagonistically to RcsB on *cupD* gene expression and on the tested RcsB-dependent phenotypes.

## Discussion

In this study we analyzed the relationship between the expression of the *cupD* gene cluster, encoding a putative fimbrial assembly system, and the activity of the adjacent genes encoding two sets of two-component regulatory systems, *rcsBC* and *pvrRS*. All these genes are located on a pathogenicity island, PAPI-1, which is carried on the genome of the highly virulent *P. aeruginosa* isolate PA14 [22]. The importance of these genes in PA14 virulence, both in animals and plants, has previously been shown by testing in frame deletion mutants [22] or transposon mutants [35]. Since *cupD* genes are likely to contribute to fimbrial biogenesis, bacterial attachment and/or biofilm formation, as previously shown with *cupA*, *cupB* and *cupC* [8], [16], we tested whether a mutation in any of these genes had any impact on attachment to surfaces. Interestingly, none of the mutations affected biofilm formation *in vitro*. Thus, if the previously described attenuated virulence phenotype in similar mutants is linked to CupD-dependent attachment or biofilm formation, it suggests that *cupD* gene expression and signaling through the *rcs/pvr* two-component systems only occur *in vivo*, or in particular environmental conditions, which were not mimicked by our *in vitro* biofilm assay.

This is reminiscent of the *cupB* and *cupC* genes, the expression of which could only be induced *in vitro* by overexpressing the RocA1 response regulator or the RocS1 sensor [9], [16]. Furthermore, when we tested *cupD* gene expression by analyzing a *cupD1-lacZ* reporter fusion, we observed no β-galactosidase activity in our *in vitro* growth conditions. Since the RcsB-RcsC two-component system is highly similar to RocA1-RocS1, we therefore tested whether *rcsB* overexpression could induce *cupD* gene expression and observed a high level of activation (>50,000 fold) of the *cupD1-lacZ* reporter fusion in these conditions. This confirms that the Rcs system positively controls *cupD* gene expression, and that the close proximity of these genes on the pathogenicity island between two direct repeats likely results from a simultaneous acquisition by horizontal gene transfer. Importantly, we also verified that the *cupD* system encodes an effective chaperone-usher pathway involved in the assembly of fimbrial structures, since CupD1 fimbriae could be specifically detected on the bacterial cell surface only upon *cupD* gene activation via RcsB. Finally, we showed that, in the same way as for CupA, CupB and CupC, assembly of CupD fimbriae resulted in increased bacterial attachment to inert surfaces and biofilm formation. In this context it is important to note that although activation of these four Cup systems results in a similar phenotype in an *in vitro* biofilm assay, the differences in regulation and organization would suggest that they are not redundant. Instead, they are likely to be specifically expressed during infection and/or within a particular niche, and each of them may therefore be responsible for specific binding to different biological surfaces, as has previously been shown for uropathogenic *E. coli* [36].

In the case of the Roc-dependent control of *cupB* and *cupC* gene expression, an intriguing antagonistic effect was observed. Whereas the RocA1 response regulator activates gene transcription in response to RocS1 signaling, the activity of RocR, the other response regulator that interacts with RocS1, was found to repress *cupB* and *cupC* gene expression [16]. PvrR shows high similarity (45%) to RocR. We thus tested whether PvrR could down-regulate *cupD* gene expression. We showed that, in the genetic background used for this experiment, *pvrR* overexpression resulted in a significant 2-fold reduction in the activity of the *cupD1-lacZ* promoter fusion. However, using a similar assay we could not show an increased activity of the fusion in a *pvrR* transposon mutant (data not shown). This could mean that efficient *cupD* gene expression requires a concomitant activation by the RcsB response regulator.

The output domains of the response regulators RocR and PvrR both contain an EAL domain rather than the classical helix-turn-helix motif. This suggests that they are not able to bind DNA and thereby directly control gene expression. Instead, their activation results in a phosphodiesterase activity, which is responsible for degradation of c-di-GMP [17]. It is now well established that low levels of intracellular c-di-GMP shift the bacterial lifestyle from sessile and biofilm-associated to planktonic and motile. We have shown that CupD fimbrial assembly is concomitant with increased biofilm formation. Furthermore, we showed that increased levels of PvrR down-regulate *cupD* gene expression and also decrease biofilm levels. This is in perfect agreement with the idea that low levels of c-di-GMP direct the bacterium towards a non-biofilm lifestyle. The overexpression of any EAL- or GGDEF-domain containing proteins could have a global effect on c-di-GMP-dependent phenotypes [30], [37]. However, the genetic linkage between *pvr* and *cup* genes strongly suggests that *cupD* gene expression is a direct target for control by the Pvr system. Our observation thus reveals that RcsB promotes CupD fimbrial assembly and biofilm formation, while activation of PvrR antagonizes this effect. This provides another parallel to the Roc system, in which RocA1 and RocR have antagonistic effects on the *cupB* and *cupC* gene targets. However, there is a fundamental difference between the two systems. The Roc system has two response regulators (RocA1 and RocR) that interact with the same sensor (RocS1), and the activation switch for the response regulators may therefore be based on their relative affinity for the sensor. In the Rcs/Pvr system on the other hand, each response regulator seems to have its own cognate sensor, RcsC or PvrS. This could indicate that the control switch between activation of RcsB or PvrR is dependent on two different stimuli, which are detected by RcsC or PvrS, respectively. However, the activation of *cupD* gene expression observed in the PA14::*rcsC* transposon mutant suggests that it may be mediated by RcsB in the absence of RcsC.

It is well documented that an elevated capacity to form biofilms tends to be associated with a number of additional phenotypes, such as decreased motility, the appearance of small colony variants, or increased autoaggregation [23], [30]–[31], [38]–[41]. For example, Drenkard and Ausubel (2002) showed that *P. aeruginosa* PA14 “rough small colony variants” (RSCV) arose as a result of antibiotic selection, and that these RSCVs exhibited increased antibiotic resistance, autoaggregation, attachment to abiotic surfaces, and formed biofilm faster [23]. In another study, small rough colonies were isolated from *P. aeruginosa* 57RP grown as biofilms or in static culture. These variants were deficient in twitching and swimming motility, autoaggregated in liquid culture and rapidly initiated the formation of strongly adherent biofilms [31]. Colony morphology variants displaying similar phenotypes have also been isolated from *P. aeruginosa* PAO1 biofilms [40]. Finally, the isolation of small colony variants from the lungs of cystic fibrosis (CF) patients [42] suggests that this morphotype may be selected for, either by antibiotic treatment or by the biofilm lifestyle that *P. aeruginosa* adopts in the lungs of CF patients.

Examination of PA14 overexpressing *rcsB*, which displays increased biofilm formation, revealed a small colony morphotype. Furthermore, in agreement with the studies mentioned above, this strain displayed reduced twitching and swimming motilities. Since one of the gene targets for the response regulator RcsB is the *cupD* gene cluster, we wished to test whether all phenotypes observed upon *rcsB* overexpression are CupD-dependent. Overexpression of *rcsB* in a *cupD* mutant resulted in either complete or partial abolition of these phenotypes. Indeed, twitching and swimming motility were only slightly affected in a *cupD* mutant overexpressing *rcsB*, and the colony size was only marginally reduced. Interestingly, overexpression of *rcsB* in a *cupD* mutant not only abolished the increased biofilm phenotype, but it further decreased the level of biofilm formation, as compared to a *cupD* mutant carrying the vector control. This suggested that whereas *rcsB* overexpression results in assembly of CupD fimbriae, in the absence of these fimbriae, RcsB activity either promotes biofilm detachment or prevents the initiation of biofilm formation. This is likely to be due to the production of another component, the activity or function of which is masked by the presence of the CupD fimbriae. Furthermore, the biofilm phenotype of the *cupD* mutant overexpressing *rcsB* is very similar to the phenotype observed when *pvrR* is overexpressed in the parental PA14 strain, which is also close to null. The ability of CupD fimbriae to interfere with the activity of other surface components was most clearly seen in the motility phenotypes. Indeed, one likely explanation for the reversion of the motility phenotypes in the *cupD* mutant could be that the presence of CupD fimbriae impaired the proper function of other cell surface appendages, such as type IV pili or flagella. A similar suggestion was made by Meissner et al (2007) for the *P. aeruginosa* small colony variant clinical isolate SCV20265, which was linked with the production of CupA fimbriae [30]. In that case, mutations in the *cupA2* or *cupA3* genes (encoding the CupA chaperone and usher respectively) reverted the bacterial behavior to non-aggregative and more motile, which could suggest that CupA fimbriae impaired swimming and twitching functions in this strain.

As mentioned above, one of the phenotypes reported for small colony variants is the autoaggregative phenotype. These variants are often named rough, wrinkled or rugose, to depict the morphology of the colony, which is believed to partly result from the increased aggregating properties of the bacteria. These variants also displayed an increased sedimentation capacity when grown in static cultures. Surprisingly, when we analyzed the sedimentation phenotype of our *rcsB* overexpressing strain, we observed that sedimentation was reduced compared to the PA14 strain not producing CupD fimbriae. We further showed that this “non-sedimenting phenotype” could be reverted by overexpressing *pvrR*, and was also lost when *rcsB* was overexpressed in a *cupD* mutant. This indicated that CupD fimbriae do not promote autoaggregation, and even interfere with the autoaggregation process. As for the motility phenotypes, we suggest that CupD fimbrial assembly could interfere with the function of other components involved in autoaggregation and/or may alter the physiochemical properties of the bacterial cell surface. This suggestion is supported by the biofilm phenotype of the *rcsB* overexpressing strain, where the biofilm capacity is not only increased, but the biofilm developmental pattern is altered. Whereas biofilm formation in the PA14 strain essentially occurs at the air/liquid interface of the culture, the *rcsB* overexpressing strain attaches to all parts of the wells, including the bottom and the walls. An important observation, which may indicate why the behavior of our colony variants differs in this respect from previously reported SCVs, is that whereas our colonies are smaller than the wild type, they do not appear wrinkly or rough, but have a smooth surface. Consequently the small colony phenotype is likely to be linked to the reduction in motility rather than to increased autoaggregation.

In conclusion, we have shown that CupD fimbrial assembly in the highly virulent PA14 strain is positively controlled by RcsB and negatively controlled by the EAL-domain containing response regulator PvrR. In addition to increased biofilm formation, several biofilm-associated phenotypes are also linked with *cupD* gene expression, including the appearance of small colony variants. This could suggest that all of these phenotypes are dependent on c-di-GMP levels, which is supported by previous observations showing that colony morphotypes and CupA fimbriae assembly are dependent on GGDEF (WspR, MorA, PA1120) and EAL-containing proteins (PvrR) [23], [30], [38]. It is also important to note that some phenotypes may not be solely dependent on CupD fimbrial assembly, but that other components controlled by RcsB and PvrR might come into play. If this is the case, they are all likely to be part of the PA14 pathogenicity islands. Indeed, we tested the phenotype of the PAO1 strain (lacking PAPI-1 and 2) upon overexpression of *rcsB*. In that case we noticed that the colony morphotype (Figure 5E and F), the motility (Figure 6E and F) and the sedimentation (Figure 7C) phenotypes were unaffected, suggesting that RcsB does not influence the *P. aeruginosa* core genome. In this context, it is also worth mentioning that although PAPI-1 contains several genes of unknown function, some of them have predicted functions, such as a gene cluster encoding a type IVB pilus assembly system and a gene encoding a pyocin. Type IVB pili may contribute to attachment and motility, and pyocin expression could select for small colony morphotypes as does for example the selection pressure imposed by exposure to antibiotics or bacteriophage [43].

Further work is required to establish the precise role of PvrR and RcsB in balancing *cupD* gene expression and to extensively characterize the RcsB/PvrR regulon and its relationship with the phenotypes presented in this study. Since all the genes of interest are located on pathogenicity islands, and the phenotypes to which they contribute to are often seen in variants isolated from the lungs of CF patients, we strongly believe that deciphering the central role of the Rcs/Pvr system in this process and characterizing the stimuli that activate these pathways is a major issue in the understanding and treatment of disease associated with chronic *P. aeruginosa* infection.

## Materials and Methods

### Bacterial strains and growth conditions

Bacterial strains and plasmids used in this study are listed in Table 2. Unless otherwise stated, cells were grown in Luria Bertani (LB) broth or on 1.5% LB agar supplemented with appropriate antibiotics at 37°C. *Escherichia coli* TG1 or OmniMAX (Invitrogen) were used for standard genetic manipulations. Transfer of plasmids into *P. aeruginosa* strains was achieved by triparental mating using the mobilizing plasmid pRK2013. *P. aeruginosa* transconjugants were isolated on Pseudomonas Isolation Agar (Difco) supplemented with appropriate antibiotics. Antibiotics were used at the following concentrations. For *E. coli*: ampicillin 50 µg/ml, gentamicin 50 µg/ml, kanamycin 50 µg/ml, streptomycin 50 µg/ml and tetracycline 15 µg/ml. For *P. aeruginosa*: carbenicillin 300 µg/ml for maintenance, 500 µg/ml for selection, gentamicin 100 µg/ml, streptomycin 2 mg/ml and tetracycline 200 µg/ml.

### Construction of plasmids and bacterial strains

Oligonucleotides for the construction of deletion mutants or overexpression constructs are listed in Table 3. Construction of clean deletion mutants was carried out as previously described [9], [44]. Briefly, mutator fragments were constructed by PCR amplification of 5–600 bp flanking each gene of interest. The fragments were joined together using SOE-PCR [45], the product was cloned into pCR2.1-TA and then sub-cloned into the *P. aeruginosa* suicide vector pKNG101. The first crossover event was selected for using streptomycin, and the second with 5% sucrose. Deletions were confirmed using external primers that were designed up- or downstream of the mutator fragments.

**Table 3:** Oligonucleotides for deletion mutants or overexpression constructs.

Deletions		Oligonucleotides (5′→3′)
***cupD***	5′ Upstream	ACATCAATCCCCTGCTCATC
	3′ Upstream	GATGTCTCAGTGCATTTCAGGGT
	5′ Downstream	CTGAAATGCACTGAGACATCC
	3′ Downstream	GCATATCGCCATTACCGACT
***rcsB***	5′ Upstream	GCAGTGGGAATGAACAGTTG
	3′ Upstream	ACTTGTCAATGGAAACGCATCAGCCCGT
	5′ Downstream	AGACGGGCTGATGCGTTTCCATTGACAA
	3′ Downstream	TACTGGTTGAACCTCTACGAAATC
***rcsC***	5′ Upstream	CTTGTACCAGCTCACTATGTTTCG
	3′ Upstream	GCATCAGCCCGTCTTCACGGGATGCGG
	5′ Downstream	CCGCATCCCGTGAAGACGGGCTGATGC
	3′ Downstream	AGTACTTCGAACTCCTTCATCGAC
***pvrR***	5′ Upstream	AGCTTTCCCCTCAGGGTGATATGGAG
	3′ Upstream	TTAAACAAGCCAGCTCATCGATCCACC
	5′ Downstream	ATGAGCTGGCTTGTTTAACGCAGT
	3′ Downstream	CACCGAGCGAAAACGGATCT
***pvrS***	5′ Upstream	GGCGGTTCGACCTGGCTCCT
	3′ Upstream	TCATCGATCCAACTTCATGCCGAAAG
	5′ Downstream	ATGAAGTTGGATCGATGAGCTGGA
	3′ Downstream	CCCGGTGTGGAGGGATTTCT
**Overexpression constructs**		**Oligonucleotides (5′→3′)**
***rcsB***	Upstream	GCGAGGTGCTCGTTCATATC
	Downstream	GCTTCAGCTGGATCGATGAC
***pvrR***	Upstream	ATCAACATGCCGAACATGAA
	Downstream	CCTCCTTCAGCGACACTGTT
***cupD1-6H***	Upstream	GGATAGCTCTTAACAGACCATTGCT
	Downstream	TCAGTGGTGGTGGTGGTGGTGCTCGTAACGCA
**Promoter fusion**		**Oligonucleotides (5′→3′)**
***cupD1***	Upstream	ACATCAATCCCCTGCTCATC
	Downstream	GTCTGGTCGGTCACTTCTCC

Overexpression constructs were obtained by PCR amplifying the gene of interest, cloning into pCR2.1-TA and sub-cloning into a pBBR broad host range vector.

The *cupD1-lacZ* fusion was obtained by PCR amplifying a fragment around 530 bp upstream and 90 bp downstream of the *cupD1* start codon, cloning the product into pCR2.1-TA and then sub-cloning into miniCTX-*lacZ*. The plasmid was transferred into *P. aeruginosa* by bi-parental mating with *E. coli* SM10 and integrated into the *att* site of the *P. aeruginosa* as previously described [29] to create an unmarked chromosomal fusion.

### Biofilm formation

Biofilm assays were carried out in 24-well polystyrene plates essentially as previously described [9]. Briefly, overnight cultures were inoculated into 1 ml M63 medium supplemented with 1 mM MgSO_4_ and appropriate antibiotics to a final OD_600 nm_ of 0.2. Four wells were inoculated from independent cultures for each strain and timepoint. Plates were incubated without shaking at 30°C, and attached cells were stained with 100 µl crystal violet (Becton, Dickinson and Company) for 10 min. Wells were washed twice with 1 ml H_2_O, and the stain was redissolved in 1 ml 50% ethanol. Absorbance was measured at 595 nm.

### β-galactosidase assays

Independent overnight cultures (n≥3) were inoculated into M63 supplemented with 1 mM MgSO_4_ and appropriate antibiotics to OD_600 nm_ 0.1, and cultures were grown at 37°C with shaking. Samples were harvested at the times indicated in each experiment to monitor OD_600 nm_ and β-galactosidase activity. β-galactosidase activity measurements were based on the hydrolysis of *o*-nitrophenyl-β-D-galactopyranoside hydrolysis using the method of Miller [46], and activity was expressed in Miller units.

### Shearing of fimbriae

Preparation of fimbriae by shearing was carried out essentially as previously described [9]. Briefly, strains were grown on 1.5% M63 agar supplemented with 1 mM MgSO_4_, 0.4% arginine and 150 µg/ml gentamicin at 30°C for 4 days. Cells were scraped off the plates, resuspended in LB with 10 mM MgCl_2_ and incubated at 4°C with gentle stirring overnight. Cells were sedimented in a microcentrifuge (5000 rpm, 10 min, 4°C). Debris was removed from the cell free supernatant by ultracentrifugation (17,600×*g*, 15 min, 4°C), protein was precipitated at room temperature with ammonium sulphate at 50% saturation and sedimented by ultracentrifugation (70,400×*g*, 1 h).

### Production of antibodies directed against CupD1

The production of CupD1 antibody was carried out as previously described [9]. Two peptides within the amino acid sequence of CupD1, CSAVVDGRTDPTVILD and LAGGETSTSYDYAVRY, were selected and synthesized (Eurogentec). Two rabbits were then inoculated with the peptides, and this was followed by three boosters 15 days, 1 month and 2 months after the first injection. The specificity of the sera was tested on whole cell extracts of *E. coli* BL21(DE3) expressing CupD1-6H (pCR2.1-*cupD1*-6H).

### SDS-PAGE and Western blotting

Cell pellets or ammonium sulphate precipitated protein was redissolved in Tris buffer (10 mM, pH 8), protein corresponding to an equal number of cells was separated on 15% (w/v) SDS-polyacrylamide gels and blotted onto nitrocellulose membranes (Whatman). The CupD1 protein was detected using the CupD1 primary antibody at 1∶750 dilution and a peroxidase conjugated goat anti-rabbit secondary antibody at 1∶5,000 dilution (Sigma). Detection was achieved using the SuperSignal West Pico chemiluminescent kit (Perbio).

### Autoaggregation assays

Autoaggregation assays were carried out essentially as previously described [32]–[33]. Briefly, cultures were grown with shaking in LB supplemented with appropriate antibiotics. Cell density was normalized to OD_600 nm_ = 2.5 with fresh medium, 10 ml of culture was then transferred to a 14 ml round bottom tube and incubated without agitation at room temperature for 24 h before image capture.

### Motility assays

Motility assays were carried out essentially as previously described [47]. Briefly, swim assays were carried out on plates containing 10 g/L tryptone, 5 g/L NaCl, 0.3% agar (Merck) and supplemented with appropriate antibiotics. OD_600 nm_ of overnight cultures was standardized, and 0.5 µl of culture was injected below the surface of the agar. Plates were incubated at 30°C overnight. Twitch assays were carried out on 1% LB agar supplemented with appropriate antibiotics. Plates were inoculated by picking a colony using a sharp sterile toothpick and stabbing to the bottom of the plates, which were incubated at 37°C for two days. The agar was then peeled off the plate, and cells were stained with crystal violet for visualization.

## Supporting Information

Figure S1 Biofilm formation of P. aeruginosa PA14 wild type and isogenic deletion mutants. A) PA14 wild type and PA14ΔcupD, B) PA14 wild type and mutants with deletions in the rcs (PA14ΔrcsB and PA14ΔrcsC) and pvr (PA14ΔpvrR and PA14ΔpvrS) two-component systems. Biofilms were grown in 24 well plates, and attachment was quantified at different times (2 to 24 hours) using crystal violet staining. M63 is a negative control containing cell free M63 medium.
(1.42 MB TIF)
