# Supplementary figures and images for: Expression of Pseudomonas aeruginosa CupD Fimbrial Genes Is Antagonistically Controlled by RcsB and the EAL-Containing PvrR Response Regulators
Source: PLoS One. 2009 Jun 23;4(6):e6018. doi: 10.1371/journal.pone.0006018 (PMC2696094; doi:10.1371/journal.pone.0006018)

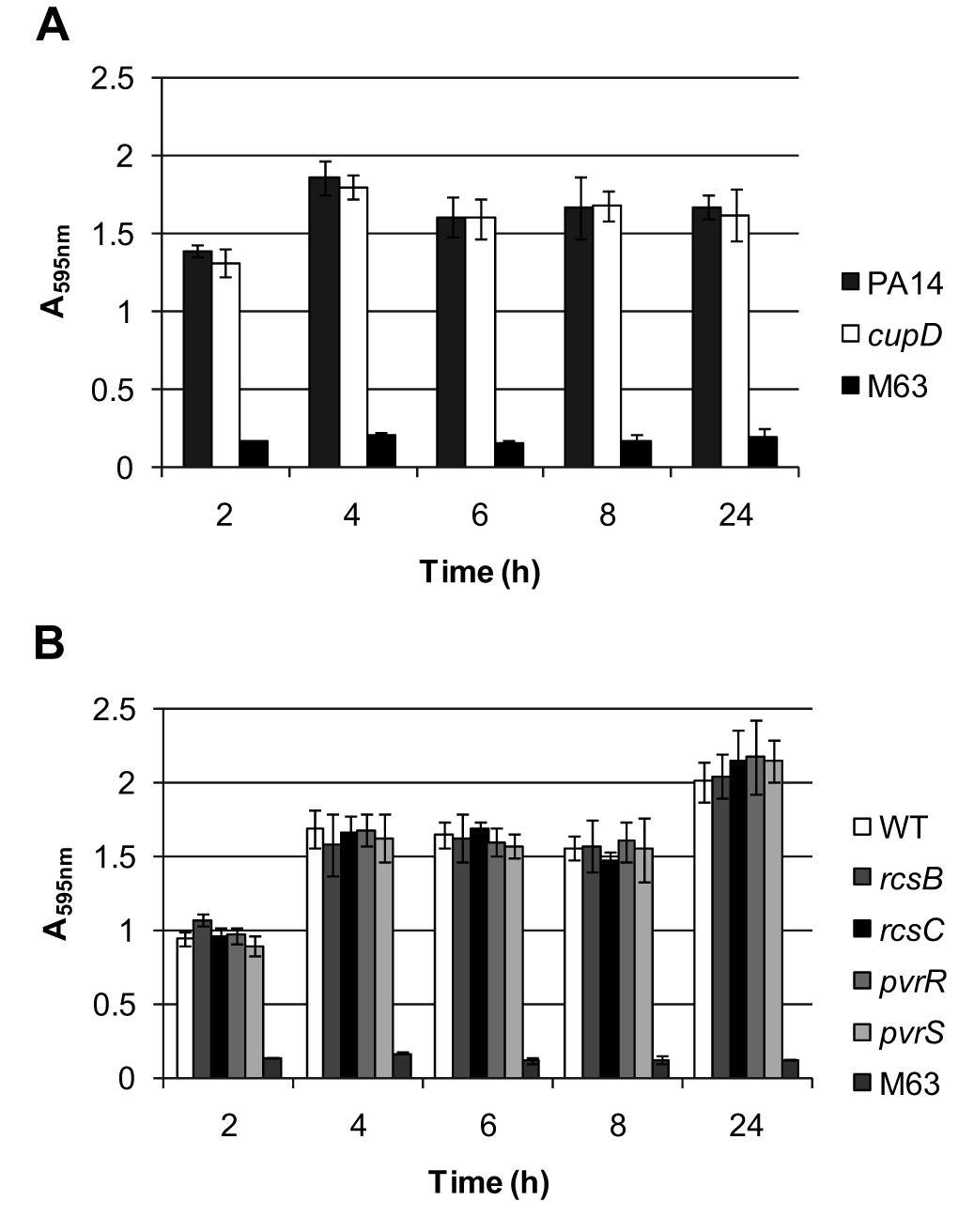

Supplement: Figure S1 — Biofilm formation of P. aeruginosa PA14 wild type and isogenic deletion mutants. A) PA14 wild type and PA14ΔcupD, B) PA14 wild type and mutants with deletions in the rcs (PA14ΔrcsB and PA14ΔrcsC) and pvr (PA14ΔpvrR and PA14ΔpvrS) two-component systems. Biofilms were grown in 24 well plates, and attachment was quantified at different times (2 to 24 hours) using crystal violet staining. M63 is a negative control containing cell free M63 medium. (1.42 MB TIF) [file pone.0006018.s001.tif]
